# Risk Assessment and Risk Minimization in Nanomedicine: A Need for Predictive, Alternative, and 3Rs Strategies

**DOI:** 10.3389/fphar.2018.00228

**Published:** 2018-03-13

**Authors:** Lisa Accomasso, Caterina Cristallini, Claudia Giachino

**Affiliations:** ^1^Department of Clinical and Biological Sciences, University of Turin, Turin, Italy; ^2^CNR, Institute for Chemical and Physical Processes IPCF, Pisa, Italy

**Keywords:** nanomaterial, nanomedicine, nanosafety, risk assessment, risk minimization

## Abstract

The use of nanomaterials in medicine has grown very rapidly, leading to a concern about possible health risks. Surely, the application of nanotechnology in medicine has many significant potentialities as it can improve human health in at least three different ways: by contributing to early disease diagnosis, improved treatment outcomes and containment of health care costs. However, toxicology or safety assessment is an integral part of any new medical technology and the nanotechnologies are no exception. The principle aim of nanosafety studies in this frame is to enable safer design of nanomedicines. The most urgent need is finding and validating novel approaches able to extrapolate acute *in vitro* results for the prediction of chronic *in vivo* effects and to this purpose a few European initiatives have been launched. While a “safe-by-design” process may be considered as utopic, “safer-by-design” is probably a reachable goal in the field of nanomedicine.

## Introduction

Nanomedicine encloses many potential promises, ranging from optimized, targeted, and even personalized treatments with decreased toxicity, to very sensitive, and cheaper diagnostic approaches with contained costs, innovative functionalized biomaterials, and the prospect of cellular and tissue regeneration strategies (Wagner et al., 2006; Senjen, 2013).

Nanotechnology-based approaches have already been translated into highly accurate and sensitive diagnostic tests, most prominent of which is the early detection of neoplastic disease (Ferrari et al., 2009), targeted therapeutic interventions, following the statement that “targeted delivery will revolutionize disease treatment” (Duncan and Gaspar, 2011) and theranostic applications, having the possibility to combine in the same disease intervention both diagnosis (through imaging) and treatment (through thermal ablation) (Lammers et al., 2011).

Nanotechnological devices used in nanomedicine possess unique properties, not found in identical devices of smaller or larger dimensions, as they stem from their nanoscale dimension (Ferrari et al., 2009). However, a consensus has not been reached yet as for a scientific definition of “nanoparticle” (NP) (Satalkar et al., 2016) and as a consequence slightly different size limit definitions are applied in different fields of nanotechnology (Schütz et al., 2013).

In this review we first summarize the current state regarding safety evaluation of nano-based therapeutics and then we focus on the growing need for nanosafety studies for safer design of nanomedicines, including the employment of novel acute *in vitro* studies to extrapolate chronic effects that occur *in vivo*.

## Risk assessment and risk minimization in nanomedicine

The implementation of nanotechnology in medicine is a process that has occurred rapidly, suddenly moving from basic research and laboratory experimentation to clinical trials (Kola and Landis, 2004; Etheridge et al., 2013; Hafner et al., 2014). Many nanomedicine formulations have already entered the market. A recent study identified 247 nanomedicine applications and products, a very significant number, approved for or nearing in-human use (Etheridge et al., 2013) and it has been envisioned that the worldwide nanomedicine market may double by 2019 (BBCResearch, 2015). In the majority of cases, these artifacts are nanoformulations of current or novel drugs (58%) or nanobiomaterials (25%); however, nanotechnology has the potential to add innovative functionality to many pharmaceutical products and medical devices (Wagner et al., 2006). As for the regulatory aspects, it remains to be determined if nanomedicines fall into the category of medicinal products or medical devices. EU legislation makes clear distinction between the two, resulting in different regulatory approaches for risk assessment of medicinal products and medical devices. In the case of medicinal products, suitable clinical trials have to be performed prior to provision of a preliminary market authorisation for human use (Directive 2001/83/EC), while in the case of medical device market introduction requires lesser degrees of testing that can vary on the basis of the risk category the device falls into [Directive 2007/47/EC currently under revision: COM (2012) 542 final].

An elevated degree of unpredictability about prospective hazards and true advantages of NPs and nanomedicines, however, created remarkable obstacles along this translational pathway (Resnik and Tinkle, 2007). As such, nanotechnological approaches have opened up a few issues with respect to their proper risk assessment and risk minimization (Hogle, 2012), with particular emphasis on human and environmental toxicity (Allhoff, 2009; Ramachandran et al., 2012). Especially compelling in this respect are “first in human” (FIH) trials of nanotechnology medical applications, as they raise the highest degree of unpredictability in all clinical area (Kimmelman and John London, 2011). Especially important for FIH nanomedicine trials is the explicit description of the study purposes within the consent form. Today, the primary and sometimes exclusive aim of FIH trials is safety (King, 2012), even though innovative technologies like nanomedicines do not always fit the classical clinical trial phases that are followed when a new drug is developed. Thus, the information contained in the consent form should focus on these purposes and emphasize all aspects related to safety testing and risk identification (King, 2012).

Research in the field of nanomaterials (NMs) applied to medicine has continued to grow upon time, but it has been primarily focused around technological improvement, and not directed toward the definition of the potential risks of nanoproducts, thus nanosafety is an area that has remained poorly assessed. The fate of a nanomaterial upon entrance in the organism, whether it will be accumulated and become toxic, or rendered available at a biological level and transformed, or if and how it will interact with cells and macromolecules inside cells, are all crucial aspects that need to be understood. The conventional approaches listed in current test guidelines are not very likely to turn out as appropriate for the assessment of nanomedicine risks, rendering it urgent to develop NM-specific standards, guidelines, and tools. It is well-known, in fact, that often the bulk materials behave different in the nanometer regime and there is the need of generally accepted test methods for the characterization of nanomaterials. The methods currently existing can be not at all suitable to characterize nanomaterials for their specific properties. For example, when nanoparticles are dispersed in water, air or biological media they show a tendency to agglomerate and can even lose their nano-dimension. For this, it is important a careful and wide morphological, physico-chemical, and *in vitro* and *in vivo* biological characterization not only on the manufactured nanomaterials but also on nanomaterials after contact with relevant media. The evaluation of the effects of dispersion methods and of molecular interaction with biological components, cells and tissues on properties of nanomaterials represent a fundamental step for an effective control of nanomaterial risk. The degradation of the nanomaterials in the biological environment, the release of molecules or debris and the functionalization with organic substances could induce cytotoxic effects to be explored using methods already employed and validated in nanotoxicology but also improving the physico-chemical characterization. *QualityNano* (www.qualitynano.eu; finished in 2015) has represented one of the first European initiatives along this line. It was an analytical research infrastructure addressing quality in NM safety assessment, through driving reliable and reproducible approaches to nanometrology and NM characterization before, during and after exposure to living systems. It included the development of standard operating protocols (SOPs) for analysis of the possible risks posed by NMs, focusing on assay reproducibility, use of appropriate positive and negative controls and controlling dose delivered to living systems (Senjen, 2013).

Assessment and management of risks, as well as risk communication are among the most challenging issues for nanomedicine clinical research (Resnik and Tinkle, 2007). Surely, our understanding and knowledge of different nanosized materials can be improved by single *in vivo* animal experiments and *ex vivo* laboratory testing, yet when a new nanomedicine product is to be tested in Phase I clinical trial they are not sufficient to resolve all of the uncertainty surrounding the first exposure of a human subject. What can be ethically accepted is that the risks potentially posed to human subjects by the new therapy are reasonable in relation to the new therapy potential benefits and, from a regulatory standpoint, that risks to the subject and society are minimized, wherever possible (Emanuel et al., 2000).

## Predictive toxicological approaches, alternative test strategies, and 3Rs approaches

Extensive preclinical and clinical testing is needed prior to application of nanomedicine products in the three relevant areas of diagnosis, prevention, and treatment of disease, yet many aspects of NMs, including their toxicological, pharmacological, and immunological properties, have entered the scientific exploration only recently. Early safety studies are exceedingly needed to define whether the risk to benefit displayed by a specific nanomedicine is acceptable for the proposed use, thus determining if that nanotechnology will have the promise for further development in a clinical application (Duncan and Gaspar, 2011). Importantly, traditional approaches to toxicology, which are inherently descriptive in nature, will need to shift to predictive toxicology and this shift ascribes to both chemicals in general and NMs in particular (Oberdorster, 2010).

- Predictive toxicology is based on mechanism-based approaches relying on high-throughput screening (HTS) techniques. (i) The starting point is the generation of *in vitro* toxicity data resulting from the application of multiparametric, automated screening procedures. This *in vitro* phase of work may predict the possibility for disease or other pathological outcomes *in vivo*, based on the specific physicochemical properties of engineered NMs that are described (Nel et al., 2013b). (ii) The *in vivo* step will first of all validate the HTS techniques and will then improve them by establishing clear structure activity relationships. (iii) Then heat maps are developed on the basis of normalized data set, and self-organizing map features are exploited to organize all these information. (iv) Finally, appropriate combinations of both *in vitro* and *in vivo* approaches can be defined, with the final goal to establish hazard ranking and modeling. The landmark 2007 report from the US National Academy of Sciences, “Toxicity Testing in the Twenty-first Century: A Vision and a Strategy” (http://www.nap.edu/catalog.php?record_id=11970) is in agreement with this operation modality, clearly defining that a transition from qualitative laboratory testing and descriptive animal studies to mechanistic, quantitative testing funded on the employment of human cell types and high-throughput approaches will dramatically increase efficiency of toxicity evaluation (Nel et al., 2013b). Hazard assessment of large numbers of NMs can in this way be performed using pathways of toxicity (POTs), consisting in mechanism-based testing and representing the aim of the predictive toxicology approach (Nel, 2013). For the successful implementation of this methodology, careful selection of HTS techniques to be used *in vitro* as well as of POTs designed at the cellular level is needed, in order for them to reflect as many as possible pathogenic effects at the organism level. Although many methods and protocols were developed and validated for a predictive risk assessment of NMs, more work is needed regarding both physicochemical properties of NMs and their interaction with biological media. For example, the surface chemistry of the particles should be evaluated with particular attention considering that dissolution/dispersion and fate of NMs in biological media are affected by particle surface, surface charge, and radical formation potential.
- Use of alternative test strategies (ATS), aimed at reducing the number of animal testing by widening the employment of *in vitro* and *in silico* strategies, represents a promising new toxicological paradigm for NMs in medicine (Nel, 2013). Existing and emerging methods used as part of an ATS are presented in Table 1. HTS techniques, high-content screening, and computational modeling are all important resources for ATS, having the potentiality to analyse in a comparative way many NMs simultaneously (Nel, 2013). Further, the use of ATS approach allows for multiple hazard assessment steps during the entire process of product development and provides large enough amounts of data to reduce the number of animals used by prioritizing testing at each of the incremental assessment stages described above (Nel et al., 2013a). As a matter of fact, numerous challenges still need to be faced prior to complete acceptance of ATS. For example, cellular HTS is still limited when a chronic disease condition is to be studied through predictive toxicological approaches, because currently *in vitro* cultured cells cannot recapitulate the chronology of the multistep process leading to a chronic disease at the organ or systemic level (Leist and Hartung, 2013). It is also not always easy to discriminate between end-points that disclose adverse outcomes, or that may conduct to adverse outcomes later on, and those that reflect non-adverse outcomes (Slikker et al., 2004). For regulatory purposes, risk assessment or risk management based on the use of ATS to replace for animal testing is not yet at the level of general acceptance. However, the potential utility of ATS approaches to investigate NM hazard is not under discussion and there is a general agreement about the application of ATS approaches to prioritize NMs for further subsequent toxicity testing and risk assessment prior to or upon product development (Nel et al., 2013a). Future strategies should include refinement of existing tests, such as development of organotypic 3D co-cultures, use of primary cells or stem-cell-derived systems and expansion of endpoints (i.e., carcinogenicity for chronic exposure) and their simultaneous testing. In addition, there is a need to predict the distribution, translocation, and bioaccumulation of NMs throughout the human body after exposure: pharmacokinetic (PBPK) models already developed in animal for specific NM are expected to be extrapolated also to humans. Finally, a comparison between *in vitro* and *in vivo* tests and long-term evaluation should be carefully considered.
- Reduction of *in vivo* experiments through the employment of alternative testing approaches is in agreement with the 3Rs rule (refine, reduce, and replace animal testing). There is an opportunity to improve regulatory toxicology by first optimizing the use of the entire amount of existing information concerning groups of structurally similar materials, by second gaining information from *in vitro* and *in silico* experimental approaches, and finally conducting targeted animal testing only when necessary (Hartung, 2009). Ideally, these types of strategies should encompass decision points that depend on *ad interim* results and a critical aspect of the validation process becomes a correlation between *in vitro* and *in silico* with *in vivo* results. From an ethical and economical perspective it is not acceptable to test each NM in animals, thus a triage step based on an *in vitro* screening of these materials is necessary. Efforts in this direction are being made in the USA (Nel, 2013), but also in Europe collaborative initiatives are being created to increase open conveyance and sharing of results between different research groups. Overall, testing programme for regulatory purpose addressed a series of physico-chemical endpoints including methods and assays for a list of manufactured NMs, mostly inorganic carbon and metal oxide NMs (TiO_2_, SiO_2_, ZnO, CeO_2_). For different physico-chemical endpoints, several methods were used to evaluate: chemical composition (assay: EDX, ICP-OES ICP-MS, CHN elemental analysis), size [assay: DLS, TEM, SEM, AFM, (U)SAXS), WAXS], shape (TEM), coating (XPS, STEM-EDS, FTIR analysis of functional groups), surface area (VSSA, SAXS), Water solubility/dispersibility, cristallite size (XRD) (Rasmussen et al., 2018). *In vitro* studies addressed cytotoxicity, immunotoxicity, and genotoxicity testing using different cell culture models (i.e., blood, lung, placenta, brain, liver, gastrointestinal system) according to harmonized protocols.

**Table 1:** Existing and emerging methods used as part of an ATS for NM evaluation.

**Test type**	**Aim**	**Key stages or assay**	**Limitation**	**Ref**
Genotoxicity	Rapid measurement of DNA damage, chromosomal damage; detection of upregulated DNA damage signaling pathways	- MN (Micronucleus assay) - CometChip Platform - γ-H2AX assay - FADU (Fluorimetric Detection of Alkaline DNA Unwinding) assay - ToxTracker reporter assay	Many factors can artificially influence assay results, as material and environment	Nelson et al., 2017
QSAR (Quantitative Structure Activity Relationships)	Prediction of nanomaterial exposure-dose-response	Steps of data assembling, structure characterization, model construction, model evaluation, and lastly interpretation of mechanisms	Small number of data sets	Winkler, 2016
SSDs (species sensitivity distributions)	Estimation of the maximum acceptable concentrations of chemicals in environmental risk assessment	Computational approaches	Few data known	Chen et al., 2017
Band gap analysis	Prediction of toxic potential using metal oxide conduction band energy levels	*In vitro* toxicological effect related to conduction energy and metal dissolution	Limited to metal based nanomaterials	Zhang et al., 2012
Cytotoxicity	Screening of nanomaterial-induced cytotoxicity	- Cellular metabolic activity - Oxidative stress - Apoptosis - Cell membrane integrity - Impedance based integrity	Time-consuming, labor-intensive, complex, and in some instances unreliable owing to NM interferences	Cimpan et al., 2013; Guadagnini et al., 2015; Accomasso et al., 2016
OMICS	Identification of new pathways and mechanisms in nanotoxicity not visible in conventional testing	1.1.1 Epigenomics—miRNomics 1.1.2 Epigenomics—DNA methylation and histone modification 1.1.3 Transcriptomics 1.1.4 Proteomics 1.1.5 Metabolomics	Request for high sample quality (freezing, protection against degradation) Lack of standardization of sample preparation Predictive value of the omics techniques not entirely clear	Fröhlich, 2017
High-content analysis	Capacity for monitoring a range of morphometric, functional, and biochemical properties of cells	Simultaneous identification of different parameters using fluorescence	Possible fluorescence-dye toxicity Limitation in adequate cell line	Brayden et al., 2015

The *NanoTEST* project (http://www.nanotest-fp7.eu/; finished in 2012) was one of the first examples. Many efforts were put in defining appropriate standard protocols, whose frequent lack represented an important problem experienced in testing NP potential hazards before clinical application (Juillerat-Jeanneret et al., 2015). A representative selection of commercial NMs currently or soon-to-be-applied in human medicine was investigated. To identify relevant short-term hazard models, the project used several standard toxicity assays for different markers such as cell viability, pro inflammatory response, oxidative stress, genotoxicity, immunotoxicity, cell uptake, and transport. Upon completion of the study, indications for full appraisal of NP toxicity included a few cytotoxicity measurements, a set of 2–3 representative cell types and five NP concentrations (Dusinska et al., 2015). *NANoREG* (www.nanoreg.eu; finished in 2017), *FutureNanoNeeds* (www.futurenanoneeds.eu; finished in 2017), and the ongoing *NanoReg2* (www.nanoreg2.eu) are three other European projects aimed at defining a customary European strategy to the regulatory testing of fabricated NMs and at evolving an innovative frame to allow proper classification, better naming as well as hazard and environmental impact assessment of the future NMs before their extensive industrial employment.

## Application of alternative test strategies to risk assessment and minimization in nanomedicine

A number of different nano-specific, well-designed ATS are under development having the potentiality to provide answers to focused NM toxicity questions (Shatkin and Ong, 2016).

When the NM under study has an unknown toxicity, adoption of a Weight of Evidence (WoE) approach can be considered. Risk assessment is determined following careful hazard identification and prioritization taking into consideration and weighting all *in vitro* data available, both qualitative and quantitative, even in the absence of animal data. WoE mainly represents a methodological approach, where a collection of studies is analyzed based on expert opinions, systematic reviews or meta-analyses. When possible, quantitative WoE evaluations are also applied (Hristozov et al., 2014), for instance for prioritizing the riskiest occupational exposure scenarios that, in the case of NMs, can include processing methods, handling methods, length of time of exposure, protective equipment. They will be all considered and weighted accordingly, on the basis of quantitative data and expert judgement. Multiple techniques to test one end point should be applied to generate data with a high enough quality to allow for regulatory decisions to be taken based on WoE approaches.

Intelligent/Integrated Testing Strategies (ITS) are applied for the identification and prioritization of nanosafety research needs (Stone et al., 2014). Through this method, hypothesis-driven questions put to make risk decisions are answered by combining existing data, available analytical tools, experimental tests where the main goal is to avoid the need to test each developed NM. This strategy has been applied to accelerate the risk assessment process for materials of concern (Jaworska and Hoffmann, 2010) and benefits include reduced testing, consequently lowering the costs and limiting animal use; possibility to categorize NMs by (potential) mechanisms of action; applicability to a large assortment of testing strategies. Generally, ITS is based on of a stepped framework beginning with (i) an evaluation of existing data, that are organized using implements such as adverse outcome pathways (see below); (ii) measurement of chemical properties; (iii) biokinetic study of the NM; (iv) choice of suitable toxicity tests; and (v) employment of a WoE analysis that takes in consideration all the above results. Refinement steps can follow concerning both strategy and methods after which WoE is reevaluated (Oomen et al., 2014).

Conceptual frameworks such as adverse outcome pathways (AOP) are also been developed. Risk assessment is here performed through a sequential chain of events that are all causally linked and lead to an adverse outcome. Based on existing data, an initial molecular event is described and linked to a series of downstream key events acting at different biological levels (organism-cell-molecule) and eventually leading to the adverse outcome (Ankley et al., 2010).

All the above-mentioned approaches are needed in order to enable alignment of nanotoxicology with the 3Rs (Burden et al., 2017). The first step, establishment of a regulatory framework to enable implementation of alternative non-animal methods into risk assessment and acceptance, can benefit from WoE approaches to consider all available evidence from different non-animal methods. This will increase regulatory confidence in results from non-traditional methods, via guidelines and appropriate training, and will support risk assessors to understand the relevance and applicability of *in vitro* data for risk assessment and to adopt a rationale to deal with uncertainties and limitations inherent to experimental models (both *in vitro* and *in vivo*). The subsequent hazard prediction step will rely on ITS and adoption of a dual approach: hypothesis driven studies which test if a particular nanomaterial property impacts on toxicity, and studies which compare the toxicity of panels of nanomaterials. These parallel approaches will aim to accurately identify which NM properties confer toxicity and to establish a “reference data” for different endpoints for NMs which are deemed “representative” (dependent on the NM being studied) and the use of appropriate positive controls to relate the effects *in vitro/in vivo*. This involves ensuring that knowledge already in existence in other areas of toxicology is utilized to build knowledge within the discipline of nanotoxicology. After a validation step, AOPs frameworks can be exploited to adapt current standard *in vitro* approaches and to improve test item preparation, dosing, and understanding of toxicity mechanisms.

A rational design of the nanomaterials from the early phase of material selection, production method optimization, and product purification has to be considered of fundamental importance to prevent the safety issues of nanomaterial and increase their applicative potential. The concept of safer-by-design emphasizes the importance of the contribution of more scientists such as engineers, chemists, physicians, and biologists to contrast the challenges of nanomaterials and satisfy the needs of the EU to regulate manufactured nanomaterials. The use of advanced analytical techniques (i.e., ICP, AFM, Chemical Imaging, biomarker detection) or their combination for the study of interactions between nanomaterials-relevant media in parallel with a better control of the preparation process will likely open up new scenarios in nanotoxicology testing (Dusinska et al., 2017; Oomen et al., 2018).

## Conclusions and remarks

In nanomedicine, a proper risk evaluation in relation to health is unavoidable, in order to safeguard societal, ethical and regulatory acceptance, and public confidence. However, the individual testing approaches are limited and have turned out to be inadequate for nanotoxicology evaluations, thus risk assessment has needed to evolve to accommodate predictive toxicological analyses and ATS. The main short- to medium-term objectives should include an improved comprehension of processes of interaction of NMs utilized in nanomedicine with organs, tissues, and cells and a clear strategy to tackle critical topics connecting to toxicity assessment specifically with respect to alternatives to tests on animals. Among the most compelling future objectives will be the need to test nanomedicines not only in healthy physiological environments, but also in disease environments that may alter biological responses and impact safety; in addition, to test nanoparticles not only individually but within complex mixtures, considering that nanoparticle incorporation into a variety of already utilized medical applications are likely to alter their risk profiles.

A safer-by-design concept has become increasingly important in risk assessment and minimization of nanomedicines, with the idea of integrating knowledge of NMs' potential adverse effects into the process of designing nanoproducts. This entails that nanomedicine safety is to be considered as an integrated route from the very first phases of research and innovation to the last phases of product validation, clearly different from the classical safety evaluation paradigm seeking to address potential concerns and to regulate NMs downstream, close to full product development, and market entrance.

## Author contributions

CC and CG contributed conception and design of the study; CG wrote the first draft of the manuscript; LA and CC wrote sections of the manuscript; LA contributed design of the table. All authors read and approved the submitted version.

### Conflict of interest statement

The authors declare that the research was conducted in the absence of any commercial or financial relationships that could be construed as a potential conflict of interest.
